# mHealth Strategies Related to HIV Postexposure Prophylaxis Knowledge and Access: Systematic Literature Review, Technology Prospecting of Patent Databases, and Systematic Search on App Stores

**DOI:** 10.2196/23912

**Published:** 2021-02-16

**Authors:** Artur Acelino Francisco Luz Nunes Queiroz, Isabel Amélia Costa Mendes, Simone de Godoy, Luís Velez Lapão, Sónia Dias

**Affiliations:** 1 Department of General and Specialized Nursing, Escola de Enfermagem de Ribeirão Preto Universidade de São Paulo Ribeirão Preto Brazil; 2 Escola Nacional de Saúde Pública Universidade Nova de Lisboa Lisboa Portugal; 3 Global Health and Tropical Medicine, Instituto de Higiene e Medicina Tropical Universidade Nova de Lisboa Lisboa Portugal

**Keywords:** HIV, eHealth, mHealth, postexposure prophylaxis, PEP, prevention, mobile phone

## Abstract

**Background:**

Globally, the number of HIV cases continue to increase, despite the development of multiple prevention strategies. New cases of HIV have been reported disproportionately more in men who have sex with men and other vulnerable populations. Issues such as internalized and structural homophobia prevent these men from accessing prevention strategies such as postexposure prophylaxis (PEP). Mobile health (mHealth) interventions are known to be one of the newest and preferred options to enhance PEP knowledge and access.

**Objective:**

The aim of this study was to identify and analyze the mobile apps addressing PEP for HIV infections.

**Methods:**

We conducted a descriptive exploratory study in 3 sequential phases: systematic literature review, patent analysis, and systematic search of app stores. For the systematic review, we followed the Preferred Reporting Items for Systematic Reviews and Meta-Analyses guidelines adapted for an integrative review in the databases of PubMed, Web of Knowledge, Scopus, Cochrane, Embase, Science Direct, Eric, Treasure, and CINAHL. The patent analysis was performed by exploring the databases of the Brazilian National Institute of Industrial Property, the United States Patent and Trademark Office, and the European Patent Office. For the systematic search, we analyzed mHealth apps related to HIV in 2 major app libraries, that is, Google Play Store and App Store. The apps were evaluated by name, characteristics, functions, and availability in iPhone operating system/Android phones.

**Results:**

We analyzed 22 studies, of which 2 were selected for the final stage. Both studies present the use of apps as mHealth strategies aimed at improving the sexual health of men who have sex with men, and they were classified as decision support systems. The search in the patent databases showed only 1 result, which was not related to the topic since it was a drug intervention. In the app libraries, 25 apps were found and analyzed, with 15 (60%) apps available for Android systems but only 3 (12%) addressing PEP. In general, the apps inform about HIV and HIV prevention and treatment, with the focus users being health care providers, people with HIV, or the general population, but they have only limited features available, that is, mainly text, images, and videos. The 3 apps exclusively focusing on PEP were created by researchers from Brazilian universities.

**Conclusions:**

Our review found no connection between the scientific studies, registered patents, and the available apps related to PEP; this finding indicates that these available apps do not have a theoretical or a methodological background in their creation. Thus, since the scientific knowledge on HIV is not translated into technological products, preventing the emergence of new infections, especially in the more vulnerable groups, is difficult. In the future, researchers and the community must work in synergy to create more mHealth tools aimed at PEP.

## Introduction

The efforts to globally fight HIV/AIDS have been increasing in the past 30 years since the first HIV global epidemic. There has been a steady global progress in the reduction of AIDS-related deaths over the last decade but a slower progress in the reduction of new HIV infections. However, epidemiological trends have remained consistent over the years, with men who have sex with men being disproportionately more affected by HIV than other populations [1].

In 2014, the UNAIDS (Joint United Nations Program on HIV/AIDS) launched a 90-90-90 target to combat AIDS as a public health threat by 2030, and the joint action of country-led and region-led efforts was required to establish this target for HIV treatment scale-up. The goal was that, by 2020, 90% of all people with HIV will know their HIV-positive status, 90% of all people diagnosed with HIV will have access to antiretroviral treatment, and 90% of all people under treatment will have an undetectable viral load [2]. According to the most recent UNAIDS data, 1.7 million new HIV infections were reported in 2019—more than 3 times the 2020 target. This incidence increased by 690,000 in 2020, indicating that we are very far from ending the HIV global epidemic [1]. A series of new tools complementary to the use of condoms and treatment as a form of prevention has been introduced to achieve the 90-90-90 goals, with emphasis on postexposure prophylaxis (PEP) and pre-exposure prophylaxis (PrEP).

PrEP is the daily use of a combination of antiretrovirals (tenofovir plus emtricitabine) to prevent the acquisition of HIV infection. The World Health Organization recommends that people at substantial risk of HIV infection should be offered PrEP as an additional prevention choice as part of comprehensive prevention [1]. PEP is short-term antiretroviral treatment that is one of the key prevention strategies in reducing the likelihood of HIV infection after potential (possible) exposure to the virus, either occupationally or through sexual intercourse, and the success rate of this strategy has been reported to be 89% [3].

PEP is of crucial importance in preventing HIV infections since it can be used even in high-risk situations, for example, during condom tear in sexual intercourse, contact with partners with a high viral or unknown viral load, and even in cases of sexual violence. The main challenge for the consolidation of PEP as one of the main preventive measures against HIV infections is the need for a specific initiation period—2 hours being considered as optimal and up to 72 hours as acceptable. Therefore, this situation is considered as an emergency [3].

The literature points out that, although crucial, data on the use of PEP are still incipient, as they are collected retrospectively instead of systematically [3,4]. Vulnerable populations for infection control are the main potential users of this strategy. However, for this purpose, they need to be properly welcomed into the health care service. There is evidence that serodiscordant couples undergoing treatment are unaware of the option of adhering to PEP for HIV prevention [4] and men who have sex with men do not identify risk situations for exposure to HIV [5].

Mobile health (mHealth) interventions have emerged as a promising tool to support disease self-management among people living with HIV, especially by promoting drug adherence and information sources. mHealth technologies have shown potential in improving patients’ communication with their health care providers by offering education and supporting the management of various chronic conditions, including diabetes, cardiovascular disease, and HIV infections. However, for mHealth interventions to be effective, they need to be developed and optimized with the needs of people in mind [6]. Despite this potential, little is known about the usage of this tool to work toward the UNAIDS goals of preventing new infections or improving PEP usage.

In light of the current COVID-19 pandemic, the prevention of HIV infections is facing unprecedented barriers such as shortage of health care professionals and resources and physical distancing from health care services. To overcome this challenge, health care systems and health care professionals are seeking new strategies, particularly eHealth and mHealth strategies, for the prevention of HIV spread [7]. Thus, in this study, we aimed to identify and analyze mobile apps that address PEP.

## Methods

### Study Design

We conducted a descriptive exploratory study [8] in 3 sequential phases: systematic literature review, patent analysis, and systematic search on app stores. An integrative review is an important resource for evidence-based practices and provides systematic techniques to summarize the literature on a given subject, thereby providing a unique view from different perspectives [9]. As a complement, studies [10] on technology prospecting refer to activities of technology determination focused on technological changes in functional capacity or on when the technologies were created and their innovativeness. This type of study aims at incorporating information into the technology management process, thereby predicting the possible future states of the technology or conditions that affect its contribution to established goals. Therefore, prospecting studies are used to determine the current state of a certain technological area and to generate information about its trajectory, future, and market trends, as well as to perceive weaknesses in certain areas [11]. In other words, these studies portray what is being recently done and can be used to point out the gaps that still need to be addressed. Thus, these studies constitute fundamental components to enhance the capacity to guide and subsidize the organization of innovation systems, transcending the business scope toward the academic environment.

### Phase 1: Literature Review

To conduct a comprehensive review, we followed the Preferred Reporting Items for Systematic Reviews and Meta-Analyses guidelines adapted for an integrative review owing to the nature of our objective and the studies related to it. We followed the following steps: (1) identifying the research question, (2) surveying relevant studies, (3) selecting the studies, (4) organizing the data, and (5) collecting, summarizing, and reporting the results [9]. Thus, we defined the mobile apps that were developed to assist in the usage of PEP as the research subject. We then used medical subject heading descriptors to search the PubMed databases through the PubMed portal of the National Library of Medicine, Web of Knowledge, Scopus, Cochrane, Embase, Science Direct, Eric, Treasure, and CINAHL by using a combination of descriptors and keywords. The defined inclusion criteria were primary studies with full text available published until July 2020, in any language. The exclusion criteria were book chapters, doctoral dissertations, master’s thesis, and technical reports in the initial search [8]. This research was conducted from June 1, 2020 to June 30, 2020 by a researcher with expertise in HIV prevention and systematic reviews. We used the following descriptors: HIV, Post-Exposure Prophylaxis, and Mobile Phone applications. Boolean operators were used to separate the keywords and descriptors (Table 1).

**Table 1:** Search strategy in each database and the selected studies.^a^

Database	Search strategy	Retrieved studies (N=22), n
PubMed	(“post-exposure prophylaxis”[MeSH Terms] OR (“post-exposure”[All Fields] AND “prophylaxis”[All Fields]) OR “post-exposure prophylaxis”[All Fields] OR (“post”[All Fields] AND “exposure”[All Fields] AND “prophylaxis”[All Fields]) OR “post exposure prophylaxis”[All Fields]) AND (“mobile applications”[MeSH Terms] OR (“mobile”[All Fields] AND “applications”[All Fields]) OR “mobile applications”[All Fields])	5
Scopus	(post-exposure AND prophylaxis AND HIV AND mobile AND applications)	3
Web of Science	(“post-exposure prophylaxis”[MeSH Terms] OR (“post-exposure”[All Fields] AND “prophylaxis”[All Fields]) OR “post-exposure prophylaxis”[All Fields] OR (“post”[All Fields] AND “exposure”[All Fields] AND “prophylaxis”[All Fields]) OR “post exposure prophylaxis”[All Fields]) AND (“mobile applications”[MeSH Terms] OR (“mobile”[All Fields] AND “applications”[All Fields]) OR “mobile applications”[All Fields])	1
CINAHL	Post-exposure prophylaxis or PEP or non-occupational post-exposure prophylaxis (nPEP) AND mobile applications	7
Cochrane	Post-exposure prophylaxis AND mobile applications	2
Embase	Post-exposure prophylaxis AND mobile applications	3
Science Direct	(“post-exposure prophylaxis”[MeSH Terms] OR (“post-exposure”[All Fields] AND “prophylaxis”[All Fields]) OR “post-exposure prophylaxis”[All Fields] OR (“post”[All Fields] AND “exposure”[All Fields] AND “prophylaxis”[All Fields]) OR “post exposure prophylaxis”[All Fields]) AND (“mobile applications”[MeSH Terms] OR (“mobile”[All Fields] AND “applications”[All Fields]) OR “mobile applications”[All Fields])	1
Eric	Post-exposure prophylaxis AND mobile applications	0
Treasure	HIV AND post-exposure prophylaxis AND mobile applications	0

^a^Source: Direct research.

**Figure 1:**
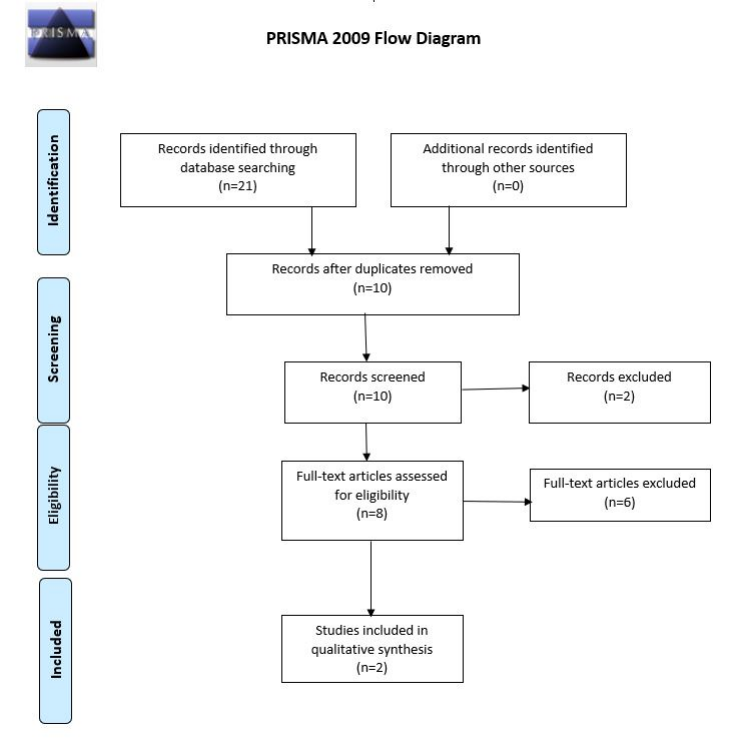
Flow diagram for the selection process following the Preferred Reporting Items for Systematic Reviews and Meta-Analyses guideline.

Two investigators conducted the analyses of the papers and discussed their inclusion during web-based meetings to reach a consensus. The titles and abstracts were read, followed by the application of the inclusion and exclusion criteria. For papers with no abstracts or if the abstracts did not permit paper exclusion or inclusion, the papers were read. The studies were analyzed, evaluating their direct relationship with the research question, along with the method, type of investigation, outcomes, objectives, sample, results, and conclusions. Duplicate studies were excluded. In total, 2 papers were selected (Figure 1).

### Phase 2: Technology Prospecting of Patent Databases

To execute this stage of the study, we focused on the countries that showed the highest production of mobile apps on PEP (based on the results of the integrative review) to define the patent databases that could be included. The patent databases that were selected were the National Institute of Industrial Property (INPI), which is the official government body responsible for the industrial property rights in Brazil, the United States Patent and Trademark Office (USPTO), which is the federal agency for granting US patents and registering trademarks, and Espacenet, which is the database of the European Patent Office, with documents from more than 90 countries. The duties of INPI include trademark registration, patent grants, technology transfer, and franchising contract registration. The search, retrieval, and export of patents on mobile apps related to PEP for HIV were carried out on July 4, 2020. In this stage, to search the INPI and Espacenet databases, structured search strategies were used with the Boolean operator “and” and the truncation to the right of type “*”. These operators were used in conjunction with the keywords to maximize the possibilities of finding the patent documents on the technology of interest. The keywords used in the searches are described below: “(HIV) AND Post-Exposure Prophylaxis AND Mobile Applications” in the “summary” field in the advanced search engine of the INPI patent database. In the Espacenet Advanced Search Builder, the keywords “HIV AND Post-Exposure Prophylaxis AND Mobile Applications” were used in the “keyword (s) in title or abstract” field. The same keywords but without the truncation features were entered in the USPTO search box.

### Phase 3: App Search

To complement the previous stage, we performed an analysis in 2 major app libraries on July 4, 2020: Google Play Store (for Android apps) and App Store (for Apple). This step enabled us to understand which apps exist, their objectives, functions, who produces them, whether these apps correspond to the published studies (phase 1), and the patents registered (phase 2). For this purpose, we used the following keywords: “HIV Post-Exposure Prophylaxis” in the Google Play Store and “HIV” and “Post-Exposure Prophylaxis” in the App Store. We found 35 apps for Android phones and 4 apps for Apple phones with these keywords. We categorized the selected apps according to the World Health Organization [12] classification for eHealth initiatives: call centers in medical care, free emergency telephone services, emergency apps for public health, mobile telemedicine services, phone reminders, community mobilization for health promotion, treatment compliance initiatives, patient records systems, systems of initiatives for information, apps for patient monitoring, mobile devices for health research, surveillance system, awareness systems, and decision support systems.

### Data Analysis

The apps were evaluated by name, characteristics, functions, and availability for the main systems (iPhone operating system or Android) (Table 2). Patent registrations were analyzed descriptively according to the information retrieved from the patent databases, titles, descriptions, and applicants/inventors. This research exempted the evaluation by an ethics committee because it did not involve human beings, according to the determinations of the Brazilian decrees 466/12 and 510/16.

**Table 2:** Distribution of the apps according to name, developer, main purpose, hosted platform, and classification.^a^

App	App Developer	Aim of app	Address postexposure prophylaxis (Yes/No)	Target population	Country	Phone operating system	World Health Organization classification
PEPtec	EEUSP Saúde Coletiva	This is an app that assists in the care of people who have experienced situations with a potential risk of HIV infection.	Yes	General population	Brazil	Android	Mobile telemedicine services
PEPusuário	EEUSP Saúde Coletiva	Assist the patient in using postexposure prophylaxis to complete the 28-day recommended measurement.	Yes	People using postexposure prophylaxis	Brazil	Android	Treatment compliance initiatives
HIV Oral PrEP Implementation Tool	Jhpiego Organization	To support the implementation of pre-exposure prophylaxis among a range of populations in different settings.	No	Care providers	United States	Android	Community mobilization for health promotion
Florida HIV/AIDS Hotline	Wellsky	Provides connection to almost 400 related HIV care services in Florida.	No	People living with HIV and general population	United States	Android	Call centers in medical care
PreP4U	HealthHIV	A resource for pre-exposure prophylaxis to prevent HIV information.	No	General population	United States	Android	Systems of initiatives for information
TánaMão	Metasix Tecnologia	This app presents an illustrative questionnaire with simple questions, which can calculate the risk rate of HIV infection.	Yes	General population	Brazil	Android	Awareness systems
EoHIV	GN1 sistemas e publicacoes	Providing information to health workers can enhance self-care and improve adherence to postexposure prophylaxis due to biological exposure.	Yes	Care providers	Brazil	Android	Systems of initiatives for information
AIDSinfo Drug Database	National Library of Medicine at National Institutes of Health	Provides information about antiretroviral drugs	No	Care providers and people living with HIV	United States	Android	Systems of initiatives for information
HIV Dating	MyDatingDirectory	Dating app for people living with HIV	No	People living with HIV	Switzerland	Android	N/A^b^
Nigeria HIV Guideline	Powered by Management Sciences for Health	Provides general and specific guidance for HIV prevention and treatment.	No	Care providers	Nigeria	Android	Systems of initiatives for information
PreParaDXS	Sociedad Española de Farmacia Hospitalaria	Information about HIV and other sexually transmitted infections, HIV testing, pre-exposure prophylaxis, and other prevention methods.	Yes	General population	Spain	Android	Systems of initiatives for information
Beat AIDS - 50+ Tips for HIV prevention	Tipsbook	Provides information about HIV prevention	Yes	General population and people living with HIV	India	Android	Systems of initiatives for information
HIV Instant Test and Guide (2019)	JOE Technologies	Information app	No	General population	United States	Android	Systems of initiatives for information
Life4me+	Life4me.plus fight to AIDS, Hepatitis C, and Tuberculosis	The app automatically remembers testing for sexually transmitted infections, taking pre-exposure HIV prevention medications, and taking antiretroviral drugs for HIV or hepatitis C.	No	General population	Switzerland	Android	Phone reminders
Jilinde	Ubunifu Ltd	To promote oral pre-exposure prophylaxis in Kenya	No	General population	Kenya	Android	Awareness systems
Sintomas do HIV	Dev Galaxy Store	Provides access to the federal government's guidelines for medical practice on HIV/AIDS.	Yes	General population	Brazil	Android	Systems of initiatives for information
2018 BHPS	National Minority AIDS Council, Inc	To inform about the third annual Biomedical HIV Prevention Summit.	No	General population	United States	Android	Systems of initiatives for information
Be-PrEP-ared	Frederik Matthesstraat	To support pre-exposure prophylaxis users in correct usage	No	General population and pre-exposure prophylaxis users	Netherlands	Android	Apps for patient monitoring
HIV and Aids	Focus Medica India Pvt. Ltd	Animated videos about HIV and AIDS	No	General population	India	Android	Systems of initiatives for information
HIV-Rx DDI Check	John Faragon	This app provides an easy-to-use Drug Interaction mobile reference based on the Department of Health and Human Services Guidelines for the Use of Antiretroviral drugs.	No	Care providers	United States	Android	Decision support systems
Guia de teste do HIV	Anadoluapps	Information about HIV	No	General population	Brazil	Android	Systems of initiatives for information
Połączenia	Program Stacja	Information about HIV	No	General population	Poland	Android	Systems of initiatives for information
RIghtTime: RI’s Sexual Health app	Rhode Island Department of Health	Offers information, resources, and videos on sexual health topics in the Rhode Island region.	No	General population	United States	Android	Systems of initiatives for information
How to Prevent HIV Infection	NonitaDev	HIV information	No	General population	United States	Android	Systems of initiatives for information
Long Exposure Camera 2	—^c^	Long-exposure photography	No	Not related to HIV content	—	Android	N/A
MSACS	IT Hubtech Solutions	To inform about Maharashtra State AIDS Control Society in India	No	General population	India	Android	Systems of initiatives for information
Pre-exposure prophylaxis	Pomorski Uniwersytet Medyczny w Szczecinie	To inform about HIV, mainly pre-exposure prophylaxis	No	General population and pre-exposure prophylaxis users	Poland	Android	Systems of initiatives for information
Yazi	Oluwatoni Fuwape	To design customized condoms	No	General population	United States	Android	N/A
EDUC@AIDS	Rvs Comunicação e Tecnologia	To inform about HIV	Yes	General population	Brazil	Android	Systems of initiatives for information
Avoid HIV and AIDS	Oualidosdev	To inform about HIV	No	General population	—	Android	Systems of initiatives for information
DIKA	DIKA	To inform about HIV	No	General population	Mozambique	Android	Systems of initiatives for information
Остров	Prometheus Studio	To create a service to increase the availability of peer counselors and support groups for people living with HIV	No	People living with HIV	—	Android	Community mobilization for health promotion
Candowell	Candowell	A social network that connects people, content, and purpose.	No	Not related to HIV	—	Android	N/A
Exposure Calculator	Evan Shortiss	Light exposure calculator	No	Not related to HIV	Spain	Android	N/A
Tratamentos e Doenças	goGOODapp	Inform about general infections	No	General population	—	Android	Systems of initiatives for information
Monthly Prescribing Reference	Haymarket Media	Inform about general drugs, including antiretroviral agents	Not	Care providers	United States	iPhone operating system	Systems of initiatives for information
ABC Medical Notes Pro for Exam	Pocketmednotes.com	Medical guide for medical students	No	Medical students	England	iPhone operating system	Mobile telemedicine services
HIV 3D study	USaMau03	Shows the HIV reference study and 3D digital cell with pins to show parts of the HIV cell structure.	No	Care providers	United States	iPhone operating system	Mobile telemedicine services
HIV Antibody Database	Zentropy Software	Display the sequence, structure, and neutralization data for neutralizing anti-HIV antibodies and some HIV-1 strains. It is meant to be a tool for scientists involved in HIV vaccine research.	No	HIV researchers	United States	iPhone operating system	Decision support systems

^a^Source: Google Play Store (2019) and App Store (2019).

^b^N/A: not applicable.

^c^Not available.

## Results

### Literature Review

The exhaustive literature review resulted in only 2 studies that guided phases 2 and 3 of our study (Figure 1), and both originated in the United States. We focused on databases used in the United States even for phases 2 and 3 of this study. The 2 studies in the literature review report intervention measures—one being a pilot study for a randomized controlled trial and the second, a full randomized controlled trial. Both studies present the use of apps as mHealth strategies aimed at improving the sexual health of men who have sex with men and can be classified as “decision support systems” by the World Health Organization standards. Still, none of the studies were related to the apps found on Google Play Store and App Store.

Our first finding in the literature review was the system created by Sullivan et al [13], the HealthMindr, which included tools that help in self-assessment, recommend prevention strategies, help in finding and ordering condoms or HIV self-tests, and send reminders for prevention services, condom use, HIV testing, and screening for PrEP and PEP. All these features were provided with information that allowed users to choose the best form of prevention that fit their lifestyles. The second finding was the “MyPEEPS” app, a peer-based system developed by Hidalgo et al and Kuhns et al [14,15] that provides educational information about HIV and sexually transmitted infections among young men who have sex with men, and it focused on raising awareness about minority stress (eg, due to sexual identity) and capacity building for condom use, emotional regulation, and negotiating interpersonal and substance-related risk; these skills were delivered through gamifications, scenarios, and role plays through 21 different activities.

### Technology Prospecting of Patent Databases

Based on the finding that most studies in the systematic review were developed in the United States of America, a systematized search was executed in the chosen patent bases as described. Only 1 patent was found in the INPI database, but it was unrelated to the subject; it described the patent for an antiviral drug, thereby suggesting the lack of technological innovation efforts in PEP.

### Systematic Search on App Stores

In total, 25 apps were retrieved, which were mostly developed for Android systems (21/25, 84%) in the United States (12/25, 48%) or Brazil (7/25, 28%). In general, of the 25 apps on HIV and HIV prevention and treatment, 8 (32%) target health care providers, 7 (28%) target people living with HIV, and 23 (92%) target the general population. The apps mainly consist of text, images, and videos (15/25, 60%). As 11 of the 25 (46%) apps were centered on information, they were classified as “Mobile telemedicine services” and “Systems of initiatives for information” [11]. Only 3 (12%) of them specifically focused on PEP (EoHIV, PEPtec, and PEPusuário), and these 3 apps were created by researchers from Brazilian universities.

EoHIV provides information for health care workers to assist in their self-care and improve adherence to PEP in the face of exposure to biological materials. The main functions of EoHIV are information provision about exposure to HIV, PEP and its side effects, planning about treatment, and provision of a calendar that enables the scheduling of the daily intake of the medication.

The focus of PEPusuário is to improve PEP adherence, thereby assisting the user to complete the 28-day treatment. This app also allows one to document the treatment start time, type of health care professional, and location. Information about the medication, its indications, and the location to access the medication are also available.

PEPtec is an app that assists people who have gone through a potential risk of HIV infection. Its design allows it to be used as a decision-making tool for receiving PEP by health care professionals in different settings such as emergency room, specialty clinic, basic health units, and maternity hospitals.

## Discussion

### Overview of the Findings

Our study combines a literature review and technological (prospecting and mobile app search) strategies to identify mobile apps for PEP. This analysis of 3 different data sources makes this study innovative in that it seeks to trace a relationship between the creation of scientific knowledge (phase 1) and its translation into technological products (phase 2 and phase 3). The lack of correspondence between the results in the 3 phases shows a gap between the construction of knowledge and its practical application.

The mapping of the mobile apps focused on PEP showed that there are very few initiatives dedicated exclusively for PEP, as most of the apps address HIV infections, with PEP being part of this broader content. The objective and content analyses of this mapping show that these apps are very similar to traditional strategies (booklets and websites) by using little or none of the wide range of tools a smartphone can offer. mHealth information initiatives are defined as services that provide access to health science publications or databases at points of care by using mobile devices. While relatively new to limited-income countries, these mHealth services are established in industrialized countries, where medical professionals are often equipped with advanced mobile devices [12]. Despite this, our data show a new trend since the exclusive PEP apps available in libraries were created in Brazil, a limited-income country. The Unified Health System in Brazil is the largest public health care system in the world, currently providing treatment and care for 150 million people. This system was part of the main HIV care network in 2018, wherein around 580,000 people living with HIV/AIDS received their antiretroviral drugs free of charge [16]. Its integration also extends to the country’s teaching and research institutions, which explains the pioneering spirit in the creation of technologies, as we have reported.

As our review shows, even though there are only few apps related to PEP, future efforts should be made not only to create mHealth services but also to ensure that these services are evidence-based and reliable. A recent survey by the World Health Organization found that only 12% of the member states reported evaluating mHealth services and little was known about how to effectively evaluate these services [12]. Mobile telemedicine initiatives include consultations between health care providers and transmission of a patient’s health-related data by using mobile devices.

The lack of innovative resources in the apps found can be a limiting factor for their use since they do not consist of attractive and engaging tools. However, one must take into consideration that more advanced mobile telemedicine initiatives require a significantly established infrastructure, fast telecommunication networks (ie, general packet radio services, 3G, 4G), and advanced technology, thereby making it challenging for growth and adoption by low- and middle-income countries [12], which have higher rates of HIV infection [1].

Governments are expressing interest in mHealth as a complementary strategy for strengthening the health care systems and for achieving health-related millennium development goals in low- and middle-income countries. This interest has taken the form of a series of mHealth deployments worldwide, which are providing early evidence of the potential for mobile and wireless technologies. mHealth is being applied in maternal and child health and in programs to reduce the burden of diseases linked with poverty, including HIV/AIDS, malaria, and tuberculosis. mHealth apps are being tested in diverse scenarios such as in improving timely access to emergency and general health services and information, managing patient care, reducing drug shortages at health clinics, and in enhancing clinical diagnosis and treatment compliance [12]. Most people using PEP or reporting knowledge of this strategy have already used it or belong to the lesbian, gay, bisexual, and transgender community (which historically has a closer relationship with HIV and its prevention forms), thereby placing a large part of the population in a window of “missed opportunity”—people who may have been candidates for this mHealth strategy but, due to lack of knowledge, could not access the service, especially heterosexual women, who are rarely contemplated by government campaigns on this subject [17].

Minority communities (by gender, sexuality, or ethnicity) are underrepresented in advertising, government, and even peer-to-peer education campaigns [18]. Evidence from a systematic review shows how lesbian, gay, bisexual, transgender, and queer + youth showed good adherence to tools tailored specifically for them. Digital health interventions have the potential to improve health disparities in this population, which in turn, would impact HIV transmission and prevention [19]. mHealth services offered to the public can help reach a larger population. However, when the applicability of the mHealth services is restricted, it becomes less attractive to the user. All the apps found in the literature are aimed and designed for men who have sex with men—a vulnerable population that is dismissed for large public health care or governmental initiatives [20]. Specially, young men who have sex with men are more prone to use eHealth and mHealth tools as ways to manage their own health similar to how they use other apps to tackle daily life issues [21]. However, there is a shortage of apps designed for other populations such as young women or transgender individuals. Evidence has shown that young people from sexual minorities, especially men who have sex with men, who perceive themselves within patterns of risky behavior are more likely to use the internet, especially on their smartphones to seek information about sexual health [22]. The combination of practicality, speed, and privacy make this tool an ideal way to deliver interventions and information. The fact that these individuals are so young is crucial in the strategic thinking of stopping the emergence of new infections; therefore, proper development of several tools based on scientific principles is critical. Despite the widespread promotion of technology-based interventions, evidence supporting their effectiveness in addressing noncommunicable diseases has not reached a consensus yet. Evaluations of apps and web-based programs as ways to deliver health interventions have reported that these technologies are no more effective than paper-based approaches or offer no additional benefit as an adjunct intervention. Although recent systematic reviews of eHealth and mHealth interventions targeting health behaviors provide some evidence of the short-term benefits, the effects are modest and long-term efficacy is yet to be established [21].

One of our main findings is that there is no correspondence between the 3 data sources assessed (scientific databases, patent databases, and mobile app libraries), which indicates that the scientific knowledge discovered and validated does not have the corresponding technology for implementation and most technologies that are available are not supported with appropriate methodological rigor.

### Limitations

Our study has the following limitations. First, even though we did a thorough search on the apps, these results did not include all the existing app libraries. However, our data are still relevant because they are obtained from App Store and Google Play Store, which are the 2 main app libraries. Second, it is possible that other apps that address this topic exist but they did not appear in our search because of the specific combination of words. We used the best possible combination to find as many apps as possible with a focus on PEP because of its specific characteristics and unique importance in HIV prevention.

### Conclusion

Our study demonstrates the lack of mHealth interventions for PEP. The apps that do exist are still focused on delivering information about HIV, and no initiative has been taken to improve access to further HIV interventions. These apps are deeply focused on the traditional format of compiling and organizing HIV information, without providing support for action. Thus, there is no available approach to prevent the emergence of new infections, especially in the more vulnerable groups (such as young lesbian, gay, bisexual, transgender) that already have high adherence to eHealth and mHealth interventions. Our review found no connection between scientific studies, registered patents, and the available apps, thereby indicating that the available apps do not have a theoretical or a methodological background in their creation. The accuracy and quality of these apps should be explored in future studies. Researchers and the community must work in synergy to create more mHealth tools aimed at PEP.

## Abbreviations

INPI: National Institute of Industrial Property
mHealth: mobile health
PEP: postexposure prophylaxis
PrEP: pre-exposure prophylaxis
UNAIDS: Joint United Nations Program on HIV/AIDS
USPTO: United States Patent and Trademark Office
